# Which Emergent Medication Should I Give Next? Repeated Use of Emergent Medications to Treat Acute Agitation

**DOI:** 10.3389/fpsyt.2021.750686

**Published:** 2021-12-07

**Authors:** Veronica B. Searles Quick, Ellen D. Herbst, Raj K. Kalapatapu

**Affiliations:** Department of Psychiatry and Behavioral Sciences, University of California, San Francisco, San Francisco, CA, United States

**Keywords:** agitation, psychiatry, emergency, sequential, repeated, medication

## Abstract

Agitation is a common symptom encountered among patients treated in psychiatric emergency settings. While there are many guidelines available for initial management of the acutely agitated patient, there is a notable dearth of guidelines that delineate recommended approaches to the acutely agitated patient in whom an initial medication intervention has failed. This manuscript aims to fill this gap by examining evidence available in the literature and providing clinical algorithms suggested by the authors for sequential medication administration in patients with persistent acute agitation in psychiatric emergency settings. We discuss risk factors for medication-related adverse events and provide options for patients who are able to take oral medications and for patients who require parenteral intervention. We conclude with a discussion of the current need for well-designed studies that examine sequential medication options in patients with persistent acute agitation.

## Introduction

Agitation is a common presenting or comorbid condition among patients in psychiatric emergency settings (1) and exists on a continuum of severity, ranging from irritability to violence (2–4). Concurrent with management of symptoms, attempting to identify the etiology of acute agitation is crucial for guiding ongoing treatment (5, 6). Recognizing and managing acute agitation effectively is essential to maintaining both patient and staff safety (7), and first-line management starts with verbal de-escalation (8).

When behavioral approaches alone are insufficient, pharmacologic interventions may be required. A significant portion of acutely agitated patients, though, do not respond to initial pharmacologic interventions (7, 9–12). An understanding of what medications may be safely administered in a sequential fashion is thus a fundamental component of acute agitation management protocols. While multiple guidelines are available that discuss first-line medications for acute agitation (7, 9, 13, 14), there is limited guidance regarding steps one should take pharmacologically when an initial medication has failed (15).

This manuscript aims to assist clinicians with managing patients with persistent acute agitation in psychiatric emergency settings by bridging the gap in the literature on this topic. Our focus is on pharmacological management of persistent acute agitation when sequential medications are needed. We discuss oral (PO), inhalational and intramuscular (IM) routes of administration, as these are most commonly available in psychiatric emergency settings. Using available evidence in the literature, we provide our own potential clinical algorithms for managing patients with persistent acute agitation and conclude with future research recommendations.

## Current Treatment Guidelines for Acute Agitation

There is a substantial body of literature reviewing safety and efficacy of medications for acute agitation from which expert consensus guidelines have been developed. A representative sample of recent guidelines are provided in Table 1, which demonstrate that even among expert bodies, there is no consensus regarding any single medication that should be used first-line to manage acute adult agitation. This variability across expert recommendations is likely driven by studies to date demonstrating that multiple approaches appear to be efficacious for managing agitation. Additionally, many current guidelines have not been revised for years and do not incorporate newer medication options, more comprehensive data regarding side effects, or the importance of certain prophylactic agents that are now considered standard of care (16).

**Table 1:** Guidelines and consensus statements regarding pharmacologic interventions for agitation.

**Guideline**	**Overview of recommendations**
*American Association for Emergency Psychiatry Project BETA Psychopharmacology Workgroup Consensus Statement* (9)	Published in 2012 and followed by many practitioners in the United States Recommendations stratified by suspected etiology of agitation: Patients with a known psychiatric disorder: PO: risperidone or olanzapine; haloperidol+benzodiazepine second-line. IM: olanzapine or ziprasidone; haloperidol+benzodiazepine second-line Authors note to avoid haloperidol+benzodiazepines if contraindications to such exist, but do not provide a clearly delineated summary of contraindications Patients with depressant intoxication: Haloperidol monotherapy (PO or IM) Avoid benzodiazepines due to risk of respiratory depression. Patients with stimulant intoxication or alcohol withdrawal: Benzodiazepine monotherapy (PO or IM) Agitation associated with delirium, where depressant withdrawal is not suspected: PO: risperidone or olanzapine; haloperidol at low dose second-line IM: olanzapine or ziprasidone, or haloperidol IM or IV, with caveat that if IV is >3 mg/day to monitor closely for EPS, and IV haloperidol requires continuous cardiac monitoring.
*British Association for Psychopharmacology and the National Association of Psychiatric Intensive Care and Low Secure Units: Evidence-based consensus guidelines for the clinical management of acute disturbance* (14)	Published in 2018 Not stratified by suspected etiology of agitation PO/inhaled/buccal recommendations: Oral-inhaled loxapine (bronchodilator should be available), aripiprazole, olanzapine, risperidone, haloperidol (baseline ECG advised), buccal midazolam and oral quetiapine. Guideline recommends against clonazepam and diazepam due to risk of adverse effects, and levopromazine given lack of evidence. Lorazepam and promethazine are described as possibly effective. IM monotherapy recommendations: Aripiprazole, droperidol (baseline ECG advised), olanzapine (avoid co-administration with benzodiazepines due to risk of sedation, respiratory depression and hypotension) Guideline recommends avoiding haloperidol monotherapy due to risk of EPS, lorazepam and diazepam due to lack of evidence, midazolam due to risk of respiratory depression, and levopromazine due to risk of cardiovascular adverse events including hypotension. IM combination therapy recommendations: Promethazine or lorazepam plus haloperidol (baseline ECG recommended) Guidelines recommend against combining promethazine with lorazepam due to lack of evidence.
*World Federation of Societies of Biological Psychiatry: Agitation Consensus* (7)	Published in 2016 Authors state it is not possible to make very specific evidence-based pharmacologic recommendations based on available studies, thus provide a series of consensus statements to be considered by practitioners: Lorazepam and first-generation antipsychotics are similar in efficacy. Agitation due to psychosis should be treated with lorazepam with an antipsychotic. Consensus also states the evidence for adding benzodiazepines to an antipsychotic is inconclusive. IM second-generation antipsychotics are not inferior to haloperidol, albeit with a different side effect profile. Agitation due to alcohol withdrawal should be treated with benzodiazepines. Agitation due to alcohol intoxication should be treated with antipsychotics. Elderly patients should be treated with lower doses. New formulations are promising (e.g., inhaled loxapine).
*Brazilian guidelines for the management of psychomotor agitation* (13)	Published in 2019 Recommendations stratified by etiology of agitation and other factors: Patients with depressant intoxication: PO: haloperidol IM: haloperidol Patients with stimulant intoxication or depressant withdrawal: PO: diazepam, clonazepam, or lorazepam IM: midazolam IV: midazolam or diazepam If agitation in these patients is severe, consider PO risperidone or IM haloperidol. Patients with psychiatric disorder: PO: risperidone, risperidone+lorazepam, asenapine, or olanzapine IM: haloperidol+promethazine, haloperidol+midazolam, droperidol, or haloperidol Patients with delirium: PO: risperidone IM: haloperidol If ETOH withdrawal: benzodiazepines Elderly patients: adjust dosage, avoid benzodiazepines
	PO: risperidone or olanzapine IM: haloperidol Pregnant patients: PO: risperidone IM: haloperidol Repeat until reaching dosing limits, then if needed, switch medication. Authors recommend avoiding IV route, and monitoring subjects before and after drug administration. Avoid antipsychotics and parenteral routes if known cardiac risk factors.

*ECG, electrocardiogram; EPS, extrapyramidal symptoms; ETOH, alcohol; IM, intramuscular; IV, intravenous; PO, oral*.

## Repeated Use of Emergent Medications: General Considerations

Table 2 highlights key factors to consider when deciding to administer sequential medication to treat persistent acute agitation. Staff members should have adequate training in identification and non-pharmacologic management of acute agitation (8, 17). Whenever a patient is being evaluated for necessity of medication, standardized measures to assess acute agitation severity should be utilized (18). Reassessment time points should be clearly defined (e.g., every 15 min), as should the goal time period to achieve calm (e.g., 45–60 min). The goal of treating acute agitation with medication should be calmness, not oversedation or obtundation from medication combinations (19, 20).

**Table 2:** General considerations when selecting emergent medications for repeated use.

Demographics	Age
	Sex
History	Allergies
	Comorbid psychiatric disorder(s)
	Comorbid substance use disorder(s)
	Comorbid medical disorder(s)
	Severity of behaviors
	Collateral information
	Unknown/1st-time patient vs. known patient
	Direct arrival from community vs. staying in facility
Objective findings	Standardized measure of acute agitation such as a rating scale
	Laboratory results such as urine toxicology
	Electrocardiogram
	Vital signs
	Height and weight
	Physical examination
Concurrent safety interventions	Level of staff training in acute agitation identification and verbal de-escalation techniques
	Seclusion
	Restraint checklist
	Restraints
	Reassessment time point
	Goal time period to achieve calm
Medication-related issues	Concurrent medications
	Drug interactions
	Pharmacodynamics, including expected time to effect
	Expected need for additional dosing
	Cumulative dosing effects
	Side effects
	Emergent medications given up to that decision point
	Prior effective emergent medications
	Pharmacokinetics including impact of medical comorbidities on drug metabolism
	Proper training of and technique by staff administering medication (especially for intramuscular route)

When a patient is unable to consent to medication, a restraint checklist should be used to reduce the risk that bias or provider limitations are impacting the decision to administer medications in an emergency setting (21). Oral pharmacological formulations are effective (22, 23) and should be utilized whenever possible prior to consideration of use of IM medications. If the IM route is indicated, staff must be properly trained in injection technique to avoid unpredictable absorption patterns and inadequate response (24).

Patient characteristics such as age and sex are important for informing treatment decisions. Certain medications are relatively contraindicated in elderly individuals due to an increased risk of adverse events in this age group (25, 26). Pediatric populations necessitate a multimodal approach with modified dosing (27), and only a subset of discussed medications are considered appropriate for use in pregnant patients (28).

A thorough history, review of medical conditions and current medications, and physical examination is important, although often not possible at the initial assessment time point due to the severity of agitation and should be reattempted when appropriate to do so. Assessing recent substance use is critical to ensure accurate diagnosis and mitigate interactions between substances and medications used to treat acute agitation.

Combining medications with similar side effect profiles may increase risk for adverse effects, and during sequential dosing, close attention should be paid to what a patient has already received to avoid or mitigate these cumulative risks. Documentation of the expected time to effect of medications already administered (29) and likelihood of requiring subsequent dosing (30, 31) can help avoid premature dosing and polypharmacy.

Current evidence from research examining first-line interventions should guide sequential medication selection (7, 9, 14, 29, 32–34). Evidence-based antipsychotics for acute agitation include loxapine, haloperidol, droperidol, olanzapine, risperidone, ziprasidone, aripiprazole, and asenapine (13, 35–53). Evidence-based benzodiazepines for acute agitation include midazolam and lorazepam (29, 32, 33, 54, 55). In the United States (U.S.), IM droperidol and midazolam are not typically used in psychiatric emergency settings unless embedded in medical emergency departments due to monitoring requirements.

Current guidelines counsel against co-administration of IM olanzapine and IM lorazepam, although some have argued the risk associated with such is overstated (56) or most relevant for patients with alcohol intoxication given elevated risk of respiratory depression in this population (57, 58). Promethazine and diphenhydramine have also been used in combination with antipsychotics to treat acute agitation (31, 42, 59). Evidence is lacking to support the combination use of a first-generation antipsychotic (FGA), anticholinergic and benzodiazepine concurrently (60). Compared to antipsychotics and benzodiazepines, ketamine has a greater incidence of adverse events when used for acute agitation (61, 62) and should be avoided in psychiatric emergency settings.

## Repeated Use of Emergent Medications: Current Evidence

There is limited evidence examining specific sequential medication options for managing persistent acute agitation, although many studies examining initial medications allowed for rescue sedation if indicated. One contributor to this limited evidence may be the United States Food and Drug Administration's restrictions on conducting clinical trials of agitation (63, 64). The majority of studies examining initial interventions for acute agitation did not evaluate associations between sequential dosing or polypharmacy and efficacy or adverse events. We summarize notable findings, while emphasizing limitations on interpretation.

In a prospective observational study of 1,403 participants receiving IM droperidol (65), 31% of participants received additional sedation including droperidol, midazolam, ketamine, diazepam and dexmedetomidine. Oversedation was significantly associated with three or more attempts at sedation (but not if droperidol was the only medication used), and with the use of benzodiazepines. Five percentage of participants experienced other adverse events (hypotension and respiratory depression were the most common).

A randomized controlled trial (RCT) of 167 participants receiving IM midazolam, olanzapine or haloperidol allowed for repeated dosing and alternative medications (66). Of participants receiving midazolam, 23% received a second dose, and 16% received alternative medication; of those receiving olanzapine, 28% received a second dose, and 11% received alternative medication; of those receiving haloperidol, 32% received a second dose, and 12% received alternative medication. There was one episode of dystonia and one fatal cardiac arrest in the haloperidol arm, the latter of which was thought partially due to stimulant intoxication.

In a prospective observational study of 2011 participants examining IM olanzapine, haloperidol, and zuclopenthixol (12, 67), ≤ 67% of participants required at least one additional dose of medication, and 62.7% received ≥2 antipsychotics during the acute phase. Participants initially receiving IM olanzapine had fewer adverse events and required fewer anticholinergics/anxiolytics. Sedation was common, and haloperidol was associated with extrapyramidal symptoms (EPS). Another observational study examining olanzapine (68) allowed for sequential dosing with additional parenteral medications. 10/489 participants receiving the IM formulation experienced respiratory depression, including five requiring intubation.

One RCT of 270 participants examining repeated IM olanzapine dosing compared to repeated dosing of IM haloperidol or placebo found that haloperidol was associated with greater incidence of EPS (69); those randomized to olanzapine 10 mg or haloperidol required fewer doses. Another RCT of 300 participants receiving IM olanzapine or IM haloperidol and promethazine found that the olanzapine group required more additional dosing (43 vs. 21%), and there were no significant between-group differences in EPS (59). An RCT of 488 participants receiving IM haloperidol, aripiprazole or placebo, allowed up to 3 injections/24 h (70); the haloperidol group had greater incidence of EPS. An RCT of 376 participants comparing sequential dosing of IM ziprasidone and haloperidol found ziprasidone was associated with fewer adverse events, and haloperidol associated with greater incidence of EPS (71).

A retrospective study of 15,918 participants received IM droperidol, olanzapine, and haloperidol examined rescue sedation requirements (31). Haloperidol was associated with greater use of rescue sedation medications. Forty nine intubations were documented; one fatality occurred in a participant who received olanzapine, haloperidol, and ketamine and was later found to have a subarachnoid hemorrhage. A prospective study examining IM ziprasidone, midazolam, haloperidol and olanzapine found 20–40% of 737 participants required sequential dosing (30). There were four intubation events, but no association between such and specific medication combinations received was reported.

An RCT of 115 participants comparing IM droperidol, lorazepam, and ziprasidone (72) and allowing for rescue sedation noted lower respiratory depression with droperidol than with ziprasidone or lorazepam. An RCT of 359 participants comparing inhaled loxapine and IM aripiprazole found that repeat administrations of both were well-tolerated, loxapine had a faster onset, and participant satisfaction was higher in the loxapine-treated group (73). An RCT of 344 participants examining repeated dosing of inhaled loxapine found that administration of two to three doses of 5–10 mg each was well-tolerated and efficacious (74). An RCT of 124 participants found sequential dosing of risperidone (total 24-h dose >6 mg) was associated with EPS (43). A retrospective analysis of 388 participants found that repeated dosing of IM antipsychotics, but not PO antipsychotics, was associated with a longer length of stay in the psychiatric emergency department (11).

Overall, while numerous studies of acute agitation management have allowed sequential medication administration, few have assessed what risks are associated with specific sequential options. Risks that have been reported include increased cardiac risk from combining QTc prolonging agents and more common known side effects with higher doses of specific agents (e.g., EPS when haloperidol is administered without an agent for EPS prophylaxis). The incidence of serious events (intubation, serious arrhythmia, or death) are quite rare even when multiple medications are used concurrently. A study of 904 participants to comprehensively assess risk factors for adverse events found that administering multiple medications within a 60-min period, alcohol intoxication, and age >65 were associated with adverse events (25), suggesting sequential medication use does increase risk of adverse events and should be used cautiously.

## Discussion: Sequential Treatment Algorithm

**Figure 1:**
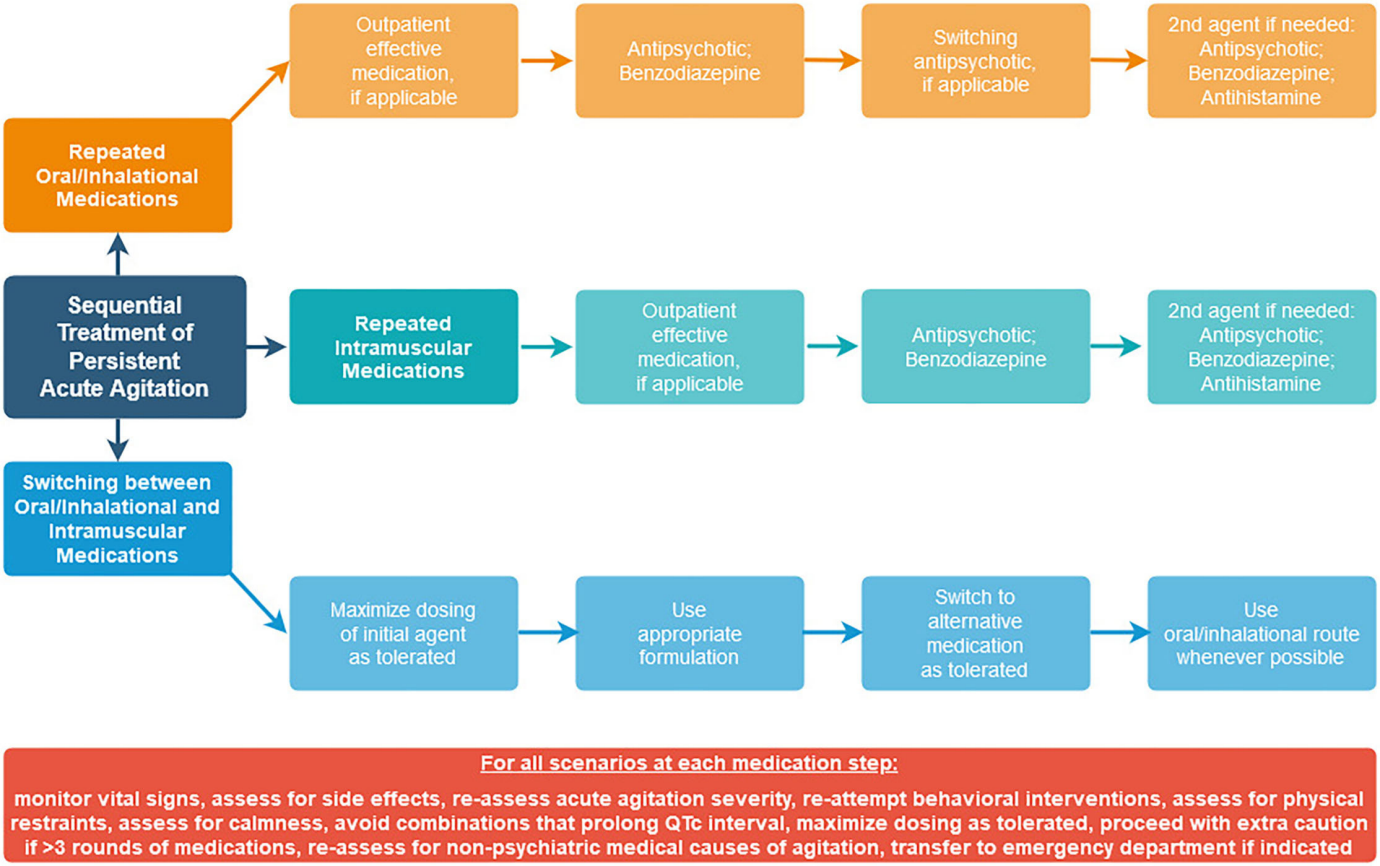
Sequential treatment algorithms for persistent acute agitation.

Since the need for guidance remains surrounding how to approach sequential medications in practice, we offer our own suggested sequential treatment algorithms (Figure 1) for persistent acute agitation that are consistent with data from available studies, using agents that are available in the United States.

Since polypharmacy is one of the main predictors of adverse events (25), an approach that focuses on limiting polypharmacy should be prioritized. Assessment of acute agitation severity using a standardized measure (18) should be completed at each time point. Behavioral interventions should be re-attempted prior to consideration of subsequent medication administration. Vital sign and side effect monitoring should be done to reduce risk of adverse events. Oral routes should be utilized over parenteral routes when possible. The goal of intervention should be to assist the patient in achieving a state of calmness, not oversedation or obtundation from medication combinations. At each step, consider expected time to onset of the medication being used, and take this into account when determining how soon another dose may be needed (32). At each step, continue to assess whether a non-psychiatric medical condition is causing or contributing to continued agitation, and whether transfer to a medical emergency department might be indicated.

### Scenario #1: Repeated Use of Oral/Inhalational Medications

This situation arises when a patient only agrees to the oral route, and acute agitation is not severe enough to justify involuntary parenteral medication administration. Which medication is most appropriate at a specific step is based on considerations in Tables 1, 2.

*Step 1*

a. If the first medication trialed was an antipsychotic, and the patient is not experiencing side effects with such, then continuing to titrate the initial medication trialed may be appropriate if there was an indication of any noticeable effect from the first dose given.
b. If the first medication trialed was a benzodiazepine, given the risk of oversedation with this class, repeated dosing of such should be approached with caution. For mild acute agitation in a patient without risk factors for a substance use disorder, and who is tolerating the initial dose well without side effects, one could consider an additional dose of the first agent used.
c. If the decision is made to switch to an antipsychotic, then olanzapine, ziprasidone, risperidone, loxapine, asenapine, and haloperidol are all reasonable options to consider with evidence to support their use, although haloperidol has a higher risk for EPS and should be administered with an agent for EPS prophylaxis. Inhalational loxapine should only be used if a bronchodilator is readily accessible and should be avoided in patients with pre-existing lung disease.
d. If it becomes known that the patient is on an effective medication as an outpatient, one may consider switching to this medication to treat acute agitation.

*Step 2*—If the patient continues to meet acute agitation criteria and re-attempts of behavioral interventions are insufficient, trial another dose of the same agent, while continuing to monitor for side effects and response. Maximize dosing as tolerated and indicated of the first agent prior to moving to Step 3.

*Step 3*

a. If the patient continues to have poor acute agitation control, sufficient time for effectiveness has been given, behavioral interventions continue to be insufficient, and acute agitation severity continues to necessitate pharmacologic intervention, one may consider another agent, guided by what has already been administered and taking into account considerations in Table 2.
b. Options include an antipsychotic (either if this was not trialed in Step 1, or if the dose of the first agent was maximized and the patient is exhibiting psychosis that would argue for continuing an antipsychotic approach), an antihistamine, or a benzodiazepine. Keep in mind the relatively higher risk for oversedation with a benzodiazepine and that with increasing polypharmacy, risk of side effects is increased.
c. Avoid combining agents that carry significant risk for QTc prolongation.
d. Maximize dosing as tolerated before moving to Step 4.

*Step 4*

a. With each additional medication administered, the risk of adverse events to the patient increases. Continue to assess acute agitation severity using structured measurements to determine whether additional medication is necessary and to reattempt behavioral interventions.
b. Consider whether physical restraints are needed to provide safety while waiting for medications to take effect, prior to increasing polypharmacy burden.
c. If >3 rounds of medications continue to be necessary, proceed with extra caution at each step while reassessing acute agitation severity, monitoring vital signs and side effects, reattempting behavioral interventions, and monitoring for calmness.

### Scenario #2: Repeated Use of Intramuscular Medications

This situation arises when a patient does not consent to oral medication and acute agitation is severe enough to justify involuntary parenteral medication. Whenever it becomes possible to do so, switch to the oral route unless the patient expresses preference for continued use of IM formulation. As in scenario #1, which medication is most appropriate at a specific step is based on considerations in Tables 1, 2.

*Step 1*

a. If the patient initially received an antipsychotic and is tolerating such, administer an additional dose of the first medication received if there was an indication of any noticeable effect from the first dose given.
b. If the patient initially received a benzodiazepine, given the risk of oversedation with this class, repeated dosing of such should be approached with caution.
c. If the decision is made to use an antipsychotic, Olanzapine IM is a reasonable choice for acute agitation management, but should not be administered within one hour of IM lorazepam. IM droperidol is also a reasonable choice, although monitoring requirements in the US limit its use in psychiatric emergency settings that are not embedded in medical emergency departments. If using ziprasidone, haloperidol or droperidol, the risk for QTc prolongation should be considered, and if using haloperidol, it should be administered with an agent for EPS prophylaxis.
d. If it becomes known that the patient is on an effective medication as an outpatient and there is an IM formulation of such, consider an additional dose of this medication *via* IM, staying within single and total daily dose limits.

*Step 2*—Follow the same approach as Step 2 in Scenario 1. Consider whether medication has been given sufficient time to take effect.

*Step 3*—Follow the same approach as Step 3 in Scenario 1. As above, note prior medications the patient has received thus far to avoid adverse events. If IM ziprasidone or haloperidol or droperidol has been used, avoid other agents with QTc-prolonging properties. Maximize dosing as tolerated prior to moving to Step 4.

*Step 4*—Follow the same approach as in Step 4 above.

### Scenario #3: Switching Between Oral/Inhalational and Intramuscular Medications

This situation arises when a patient initially agrees to the oral route as described in Scenario #1, but then declines the oral route (or becomes unable to tolerate such), or when a patient who is initially declining an oral medication (as described in Scenario #2) becomes amenable to a PO route.

In each case, if the patient has not maximized the dose of the initial agent received, and there is an appropriate formulation of such, continue to use this medication. If the patient has reached the maximum daily dose, or the medication is not available in the formulation needed, switch to an alternative medication. As in the previous scenarios, which medication is most appropriate is based on considerations in Tables 1, 2.

## Conclusion

This paper aims to fill a gap in the acute agitation literature regarding how to treat persistent acute agitation in psychiatric emergency settings when an initial medication has failed. Given the paucity of studies in this area, it is not possible to provide a single best-practices algorithm for management. The suggestions we put forth are our own clinical suggestions and are created from available data gleaned from studies of initial medication interventions for acute agitation. The general principle of using the lowest effective dose (starting at low doses and providing adequate time to effect between doses) applies, as well as incorporating new information as it becomes available to guide treatment. Aiming to achieve a state of calmness, but not oversedation or obtundation from medication combinations, should be prioritized as additional medications are added over a multi-hour timeframe. Similarly, assessment of vital signs, side effects, treatment response, and utility of behavioral interventions should be undertaken at each time point that a sequential medication is being considered. Future research in this area is clearly needed, including formal testing of repeated medications using explicit algorithms (e.g., re-dosing with the same medication vs. switching medications), with subsequent comprehensive analyses of associations between specific sequential options and both treatment response and adverse events.

## Author Contributions

VS completed the background literature search and wrote the first draft of the article. All authors have approved the final article.

## Funding

VS was supported by the National Institute of Mental Health grant R25MH06048.

## Author Disclaimer

The authors alone are responsible for the content and writing of this article. The views expressed in this article do not represent the views of the Department of Veterans Affairs or the U.S. government.

## Conflict of Interest

The authors declare that the research was conducted in the absence of any commercial or financial relationships that could be construed as a potential conflict of interest. The reviewer SZ declared a shared affiliation, with no collaboration, with the authors VS, EH, and RK at the time of the review.

## Publisher's Note

All claims expressed in this article are solely those of the authors and do not necessarily represent those of their affiliated organizations, or those of the publisher, the editors and the reviewers. Any product that may be evaluated in this article, or claim that may be made by its manufacturer, is not guaranteed or endorsed by the publisher.
